# The Influence of DNA Configuration on the Direct Strand Break Yield

**DOI:** 10.1155/2015/417501

**Published:** 2015-06-01

**Authors:** M. A. Bernal, C. E. deAlmeida, S. Incerti, C. Champion, V. Ivanchenko, Z. Francis

**Affiliations:** ^1^Gleb Wataghin Institute of Physics, State University of Campinas, 13083-859 Campinas, SP, Brazil; ^2^Radiological Sciences Laboratory, State University of Rio de Janeiro, 20550-900 Rio de Janeiro, RJ, Brazil; ^3^Centre d'Etudes Nucléaires de Bordeaux-Gradignan, University of Bordeaux, 33175 Gradignan, France; ^4^Ecoanalytica, Moscow 119899, Russia; ^5^Geant4 Associates International Ltd., West Yorkshire HX7 7BT, UK; ^6^Department of Physics, Faculty of Sciences, Saint Joseph University, Beirut 1107 2050, Lebanon

## Abstract

*Purpose*. To study the influence of DNA configuration on the direct damage yield. No indirect effect has been accounted for. *Methods*. The GEANT4-DNA code was used to simulate the interactions of protons and alpha particles with geometrical models of the A-, B-, and Z-DNA configurations. The direct total, single, and double strand break yields and site-hit probabilities were determined. Certain features of the energy deposition process were also studied. *Results*. A slight increase of the site-hit probability as a function of the incident particle linear energy transfer was found for each DNA configuration. Each DNA form presents a well-defined site-hit probability, independently of the particle linear energy transfer. Approximately 70% of the inelastic collisions and ~60% of the absorbed dose are due to secondary electrons. These fractions are slightly higher for protons than for alpha particles at the same incident energy. *Conclusions*. The total direct strand break yield for a given DNA form depends weakly on DNA conformation topology. This yield is practically determined by the target volume of the DNA configuration. However, the double strand break yield increases with the packing ratio of the DNA double helix; thus, it depends on the DNA conformation.

## 1. Introduction

The mechanisms by which ionizing radiation induces damage in DNA are very complex and multifaceted, going through physical, physicochemical, and biological stages, from the chronological point of view. This damage may lead to various biological effects, from the cellular to the organic level. Thanks mainly to the vertiginous growth in computation power during the past two decades, simulation approaches have become a powerful tool to study very complex phenomena. They have been applied to the radiation-DNA interaction process, which has been studied by combining a Monte Carlo code for the radiation transport simulation, a genetic material geometrical model, and a biophysical model to mimic DNA damage induction after a particle interaction (see [1, 2] and references therein). In atomistic DNA geometrical models [3], the volume of the target to be impacted by the radiation to induce a strand break is defined by the union of all the atoms making up the sugar-phosphate groups. This target definition will be used throughout this document. In this case, the atom size can be estimated by the corresponding van der Waals radius.

Several geometrical models used to simulate the interaction of ionizing particles with DNA have been published. A short review of these models can be found elsewhere [4]. Friedland et al. [5] have developed the most sophisticated geometrical model presented thus far. However, some model parameters are adjusted to reproduce observed damage yields for ^60^Co radiation (see [6]). The model is then applied to other radiation qualities. The effective target volume in that work is the union of all the atoms that form the sugar-phosphate group. This volume could be slightly different for each DNA form, according to the preliminary results we have obtained using the corresponding DNA structures with atomic resolution.

There are three main DNA configurations: A-, B-, and Z-DNA [7]. B-DNA is the canonical and predominant presentation of this nucleic acid, and its morphology was first described by Watson and Crick [8]. The A-DNA conformation is thought to be related to DNA-drug and DNA-protein interactions and may share with the B-DNA form the responsibility for genome structure and function (see [9] and references therein). Very recently, Whelan et al. have reported an important amount of reversible A- to B-DNA transitions in live bacterial cells by the use of Fourier transform infrared spectroscopy [10]. These authors also mention that A-DNA may be involved in the resistance of some bacteria to the damage induced by UV radiation. In contrast to A- and B-DNA, Z-DNA is a left-handed double helix macromolecule. It appears during certain physiological cellular processes and decays in B-DNA [11]. The human genetic material has approximately 100 000 copies of potential Z-DNA sequences [12]. It has also been observed that these Z-DNA sequences induce genome instability that produces DSB in certain human tumors and that they might be related to transcription activation, which is related to gene expression (see [13] and references therein). In this sense, a series of works [14–16] has noted that the structure of DNA and its binding to other macromolecules play an important role in the radiosensitivity of DNA due to the attack of OH^•^ radicals. However, these works only addressed the indirect DNA damage caused by this chemical species and did not study the relation of the direct damage and the DNA structure. Although the indirect effects play a major role in the DNA damage, their importance decreases as the LET of the incident particles increases, decreasing from approximately 65% of the damage for ^60^Co radiation (LET~0.4 keV/*μ*m) to approximately 50% for a LET of 70 keV/*μ*m (see Figure 2 of [6]). We did not account for this kind of damage in the current work as we preferred to leave this issue until our atomistic DNA models are ready for use. Atomistic models would allow a more rigorous treatment of the indirect effects [5].

Semsarha et al. [17] have recently published a study on the influence of DNA conformation on strand break yields after ^60^Co irradiation, including direct and indirect effects, although the latter has been roughly accounted for. They included the A-, B-, and Z-DNA forms and also determined microdosimetric quantities such as the mean specific imparted energy. In fact, they used a previously calculated ^60^Co electron spectrum to irradiate the region of interest uniformly instead of using the corresponding primary photon beam. Many experimental works have reported damage yields after the impact of ionizing radiation, but the difficulties associated with the determination of the precise DNA conformation during such experiments do not allow these yields to be obtained as a function of this conformation. This issue is one of the reasons we do not have any experimental reference for comparison to the results of this work.

In a previous work [19], the physical causes of the total direct strand break yield invariance with respect to the incident particle type and energy were investigated. In that work, a B-DNA representation was modeled, and it was found that this behavior results from the combination of quasiconstant number of inelastic events per unit absorbed dose and site-hit probability. This probability accounts for the chance that a sugar-phosphate group has to be hit by an energy deposition event. Furthermore, the site-hit probability seems to be determined by geometrical factors associated with the DNA model. However, only one DNA configuration was used in that study, and thus the influence of DNA conformation on the site-hit probability could not be investigated. Most of the DNA geometrical models developed to determine direct effects on DNA by Monte Carlo simulations are based on the B-DNA form (see, e.g., [3, 20]).

This work is intended to study the influence of the DNA configuration on the site-hit probability when protons and alpha particles impact the DNA. In addition, the direct total, single, and double strand break yields are also determined and analyzed. Some aspects involved in the process of energy deposition by charged particles are also studied. The differences between the volumes of the sugar-phosphate groups for the A-, B-, and Z-DNA forms have been enhanced somewhat arbitrarily to study how these volumes influence the damage yields. These differences should be large enough when relatively compared to the uncertainties associated with the determined quantities. Thus, the damage yields reported in this work should not be treated as absolute values nor be compared to experimental or previous simulated analog results. We simply want to understand how DNA conformation influences these yields.

As far as we know, this study is the first investigation of the influence of DNA configuration on the direct damage probability due to ion impact.

All the uncertainties reported in this work represent one standard deviation of the mean.

## 2. Methods and Materials

### 2.1. The GEANT4-DNA Package

The GEANT4-DNA package (v.9.4) [21] has been used to simulate the charged particle transport in liquid water. Details on the latest developments of this package can be found elsewhere [22]. Ionization, excitation, and charge transfer processes have been accounted for during the interaction of the nonnegative charge states of hydrogen and helium projectiles with liquid water. Ionizations, excitations, and elastic scattering were taken into account for electrons. Heavy charged particles were transported down to 1 keV and electrons down to ~9 eV.

### 2.2. DNA Geometrical Model

**Figure 1:**
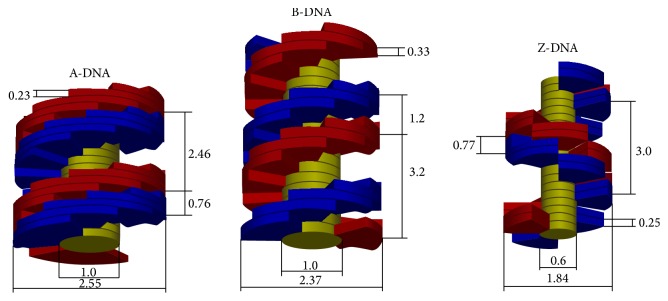
3D drawing corresponding to 20 bp fragments of the A-, B-, and Z-DNA configurations according to our geometrical model. The main dimensions of the DNA double helix are shown (in nm; see Table 1 for completeness). The figure has been slightly scaled down in the vertical direction to include 20 bp of the DNA segment.

The three main DNA geometrical configurations (A, B, and Z) were studied in this work. The B-DNA geometrical model developed in a previous work [18] was adapted to the other two variants. It is worth noting that this model accounts for five organization levels of the human genetic material: nucleotide pairs, the double helix, nucleosomes, and the 10 nm and 30 nm chromatin fibers.  Figure 1 shows a 3D drawing of DNA fragments corresponding to these configurations, according to our geometrical model. The detailed structures of the nucleosome and chromatin fibers can be found in [18]. Briefly, the nucleosome is constructed by wrapping two DNA loops around an imaginary cylinder that represents the histone. Then, the 30 nm chromatin fiber is conformed by arranging 6 nucleosomes per chromatin axial level. A nucleotide pair is conformed by two bound nitrogenous bases, which are represented by yellow cylinders in our model, with two sugar-phosphate groups attached, which are the red and blue volumes shown in Figure 1. This combination is what we call a base pair. The Z-DNA double helix is left-handed, unlike the A- and B-DNA helices. In addition, the smallest periodical structure in the latter two configurations is one base pair (bp) while in the Z configuration it is two base pairs [7].  Table 1 shows the main dimensions of the DNA molecule for the three configurations in question. Most of these data have been taken from [7]. All the three configurations present the same number of nucleosomes (2.7 × 10^6^), and each contains a number of bp that can be accommodated into two toroidal loops, maintaining a fixed external nucleosome diameter of 10.5 nm. Thus, the number of bp per nucleosome is determined by this diameter and by the bp axial step. It has been reported that the Z-DNA form cannot bend enough to form nucleosomes due to its high stiffness [23] but we decided to keep all the involved DNA conformations disposed following the same geometrical model to reduce the number of parameters that could influence damage yields. In short, all DNA forms should be organized in the same way. It should be noticed that the bp height is less than the bp axial step to avoid adjacent target overlapping due to the bending of the DNA double helix around the histone (see Table 1). It should be recalled that the targets to be impacted to produce a strand break are supposed to include all the atoms contained into a sugar-phosphate group. They have been modeled here as an angular sector of a hollow cylinder [18]. The minimum and maximum radii of these sectors correspond to the bp and the DNA helix, respectively. We adjusted the number of bp to maintain the same nucleosome radius for all three DNA configurations. Different sugar-phosphate group volumes were used to account for variations of this parameter as a function of the DNA configuration. As the uncertainties associated with the strand break yields are relatively high, we were induced to enhance the possibly different sugar-phosphate group volume for each DNA conformation. This procedure should allow resolution of the influence of this volume on the site-hit probability and the damage yield. All the regions in this geometrical model contain water with a density of 1.06 g/cm^3^.

### 2.3. Simulations

Proton beams with energies of 0.5, 1.0, 5.0, 7.0, and 10 MeV were studied in this work, as well as alpha particles with energies of 2.0, 5.0, 7.0, and 10.0 MeV. For protons, the lower energy was chosen to cover the entire region-of-interest (ROI) length with the particle range. This ROI is the same as the one defined in [19]. It is a hollow cylinder where 900 fragments of the 30 nm chromatin fiber have been axially arranged. Its central diameter is 10 *μ*m, and it is 5.25 *μ*m high. Each chromatin fragment has 500 levels containing 6 nucleosomes each. The accompanying code has now been defined to avoid a problem when analyzing hits near the 360-degree angular position in the helix reference system. This improvement led to a slight increase in the site-hit probability when compared to the results reported in [19]. For alpha particles, the minimum energy was selected in such a way that these particles had energies inside the ROI greater than the value defined by the Massey peak (~0.7 MeV). This result means that the alpha particles would have stopping power into the ROI with a defined monotony (in this case, increasing stopping power during the slowing down process). Under these conditions, the average stopping power across the ROI would have a more precise physical meaning. With these energies, the LET in the middle of the ROI for protons and alpha particles ranges within 4.8–66.9 keV/*μ*m and 58.0–235.0 keV/*μ*m, respectively. With this energy choice, the proton and alpha particle LET ranges overlap so that the strand break yield for two different particles at the same LET can be investigated.

As in our previous work [19], ion beams impact the surface of a semi-infinite water phantom in which the ROI is placed at 2.6 *μ*m depth, with its axis normal to the phantom surface. This practice is a common one when irradiating cell cultures with heavy charged particles [6]. Primary particles impinge the phantom normally and are uniformly distributed within the annulus defined by the projection of the ROI on the phantom surface. A single strand break (SSB) is recorded if an event occurs within a target, defined previously, with an energy transfer of ≥8.23 eV. As the minimum possible inelasticity during inelastic events (ionization, excitation, and charge transfer) is 9 eV in this GEANT4-DNA version, any event can potentially cause a SSB because energy transfers are always greater than or equal to the inelasticity of the reaction.

Thus, the strand break yields determined in this work must be analyzed from a relative point of view. That is, the intent is not to calculate absolute yields but to study these quantities for a few DNA configurations as a function of the beam LET. As before, a double strand break (DSB) is accounted for if two SSBs are produced on opposite helices and separated from each other by no more than 10 bp. Complex DSBs have not been accounted for in this work, and this kind of damage could be counted as two adjacent DSBs. It should be noted that the aim of this work is not to determine absolute damage yields but to study how these yields are influenced by the DNA configuration. If one target is impacted *n* times, then *n* SSBs are counted because we want to study the target-hit probability as a function of the incident and the secondary particle type and energy. The total strand break (TSB) yield is determined by using the total number of single strand breaks induced, including those leading to DSBs. The number of inelastic events due to primary and secondary particles was calculated, from which the fraction of the number of events and the absorbed dose due to electrons were found. The site-hit probability was determined as the ratio between the total number of strand breaks and the total number of the inelastic events. This probability was studied for the three DNA configurations shown above as a function of the particle type and LET.

The number of histories simulated for each particle-energy combination was chosen in such a way that the absorbed dose within the ROI was close to 100 Gy. In our previous work [19], strand break yields and site-hit probabilities were determined for a fixed dose value, that is, 100 Gy. Following this procedure, the history of the last primary particle, which would reach this dose value inside the ROI, was cut so that a portion of the primary and/or secondary particle events were not accounted for during these calculations. This cut increased the uncertainties of the quantities in question. However, these quantities are now determined by completely following, including all secondary electrons, a fixed number of histories that would amount to an absorbed dose close to 100 Gy. The number of simulated histories ranges from 4 to 200, corresponding to the 2 MeV alpha particle and 10 MeV proton cases, respectively.

## 3. Results and Discussion

**Figure 2:**
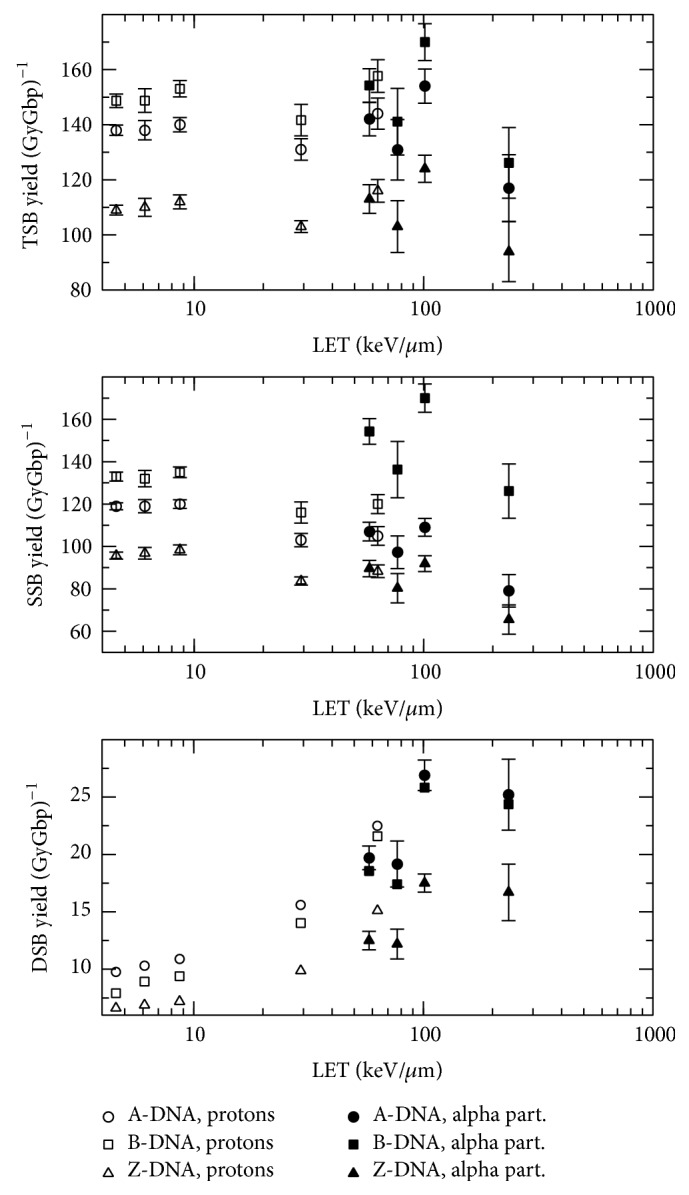
Direct total, single, and double strand break yields as a function of LET for protons and alpha particles impacting on A-, B-, and Z-DNA configurations.

**Figure 3:**
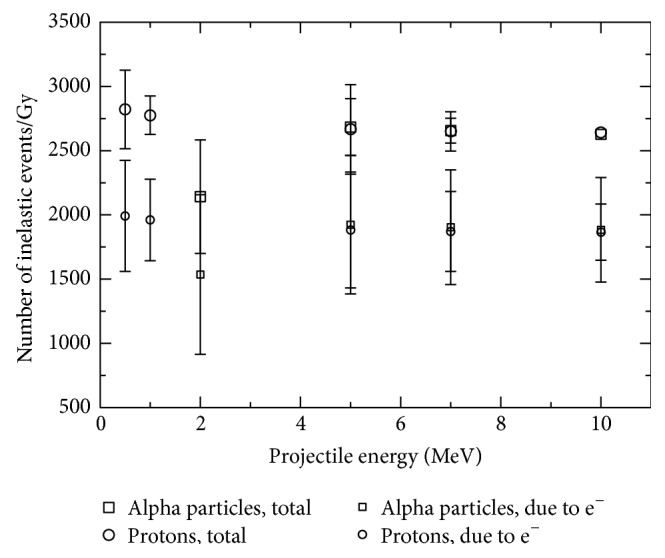
Number of inelastic events per unit absorbed dose produced by protons and alpha particles and their corresponding secondary electrons.

**Figure 4:**
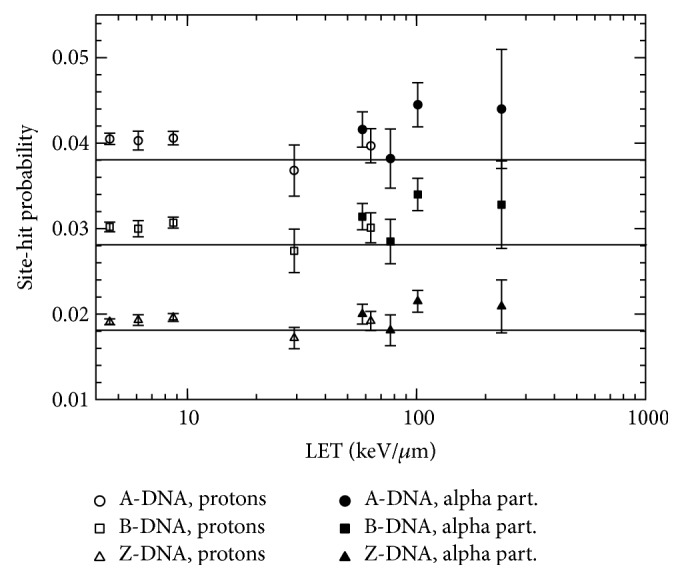
Site-hit probabilities for the three DNA configurations studied in this work, as a function of the incident beam LET.

Figure 2 shows the direct SSB, DSB, and TSB yields obtained for the A-, B-, and Z-DNA configurations as a function of the incident beam LET. It can be noted that the TSB yield for a certain DNA configuration depends weakly on the incident particle LET, remarking that the LET range is very wide. However, it can be observed that, for the 7 MeV alpha particle case (LET~100 keV/*μ*m), there is an appreciable increase in the TSB yield for all the DNA conformations. As we will see below, this result is caused by an increase of the site-hit probability. In addition, while there is also an increase in this probability for the 2 MeV alpha particle case, a decrease in the number of inelastic events per unit absorbed dose (see Figure 3) produces a relatively low TSB yield for this case. The TSB yield seems to be determined, at first order, by the target volume (see Table 1); at least it follows the same order as the target volume for all three configurations (see discussion on Figure 4). The greater the target volume the greater the TSB yield, keeping in mind that this yield is calculated per unit bp. However, the DSB yield increases with the beam LET, which is a well-known phenomenon due to the increase in the inelastic event clustering. In other words, there is a higher probability for high LET particles to produce SSBs close enough (≤10 bp) to induce DSBs. Unlike the TSB yield, the DSB yield seems to be related to the number of bp per unit helix length. For a given event cluster size and extension, which is related to the LET, the DNA conformation with the highest linear bp density would exhibit the highest probability of producing a DSB, as seen in Figure 2 (see also Table 1 for the linear bp density, i.e., the number of bp per nucleosome). It is expected that the SSB yield decreases with the beam LET for increasing DSB and quasiconstant TSB yields, but fluctuations are observed for very high LET values due to the interplay of the site-hit probability and the number of energy deposition events per unit absorbed dose. There is a kind of saturation in the DSB yield for the 2 MeV alpha particle case but this saturation is due to a relatively low number of inelastic events per unit absorbed dose for this radiation quality, which will be discussed below (see Figure 3). It is noteworthy that Friedland et al. [6] also reported a saturation in the total DSB yield, including indirect damage, for heavy ions with LET above 200 keV/*μ*m.

It should also be noted in Figure 2 (bottom graph) that the DSB yields for the two lowest alpha particle LET values are statistically similar, which occurs for all three DNA configurations. In addition, the direct DSB yield for protons is higher than the yield for alpha particles at similar LET values. As clustered ionizations are more efficient in causing DSB than sparsely distributed ionizations, it could be deduced from these results that light ions would be more efficient for producing ionization clusters than heavier particles at the same LET. This result was also found in a previous work [24].

In Figure 3, the total number of inelastic events per unit absorbed dose and that involving only secondary electrons are displayed. The total number of events is practically constant, within the uncertainty, except for the 2 MeV alpha particle case as is the number of events involving secondary electrons. Then, the fraction of the total number of inelastic events occurring within the ROI corresponding to electrons is independent of the incident particle type and energy (or LET). This result could be explained by the weak dependency of the mean energy of the electrons produced by light ions on the ion LET (see [19, 25]). In other words, secondary (first generation) electron spectra for different primary particle LET values are similar, at least for projectile energies above a few hundreds of keV/u. The differences are mainly due to the maximum electron energy, for which the spectral frequency is generally a few orders of magnitude lower than the value corresponding to low electron energies (~10 eV). The absorbed dose is calculated by adding those energy deposits made by particles within the volume in question, and thus, it could be expected that, for a quasiconstant number of events per unit absorbed dose, the mean energy deposit per inelastic event ($\bar{ɛ}$) would depend little on the particle type and energy (or LET). In a previous work [26], it was shown that $\bar{ɛ}$ is quasiconstant for protons and alpha particles impacting on liquid water, increasing by approximately 20% when the projectile energy goes from 10 keV to 10 MeV, that is, increases by three orders of magnitude. For electrons this quantity depends somewhat more strongly on the particle energy than for ions. However, the mean energy of the secondary electrons directly produced by ion impact is approximately 50 eV and $\bar{ɛ}$ shows a little change when electrons slow down from this energy to approximately 9 eV. These points are the physical reasons for the quasiconstant primary and secondary number of events per unit absorbed dose.

The site-hit probability for all the DNA configurations and for each particle energy and type was determined by dividing the total number of strand breaks by the number of targets. The results of these calculations are shown in Figure 4, in which it is observed that the site-hit probability fluctuates around a well-defined value for each DNA configuration. However, there is an increment in this probability as the particle LET increases. To study this behavior, the site-hit probability was also determined by distributing events uniformly through the ROI. That is, the positions of the energy deposition events were randomly distributed inside the ROI. In this situation, the site-hit probabilities for the A-, B-, and Z-DNA conformations were 0.038 ± 0.001, 0.0285 ± 0.0009, and 0.0184 ± 0.0006, respectively. These probabilities should be equivalent to the ratio of the volume occupied by all the targets and the volume defined by the ROI. These ratios are 0.038, 0.029, and 0.018 for the A-, B-, and Z-DNA models, respectively. These results represent a solid consistency check on our geometrical models and the associated computational codes. Lines have been drawn in Figure 4 to represent these theoretically predicted site-hit probabilities. These ratios and the average site-hit probabilities determined for each DNA configuration and primary particle are shown in Table 2. According to these results, the site-hit probability is determined by the volume ratio defined just above, at first order. The total volume occupied by all the targets is simply the target volume times the number of targets in the ROI. Thus, the important feature for the site-hit probability is the target volume, which is the sugar-phosphate group in this case. As shown in Figure 4, practically all the site-hit probabilities are above the predicted theoretical value for each DNA conformation. This fact can be attributed to the clustering of the energy deposition events. It should be noted that this overestimation tends to increase as the LET of the incident particles increases, where clustering is enhanced.

It seems that the conformation of the DNA has little influence on the total strand break yield; instead, the target volume is the key factor. However, this conformation is important for the DSB yield, where the distance between adjacent bp plays the main role. In conjunction with the quasiconstant number of events per unit absorbed dose, this point leads to the quasiconstant TSB yield shown in Figure 2. In a real DNA structure, the volume of the target would be determined by the radius of the corresponding atoms, which can be taken as their van der Waals radii, to a first approximation. In this case, a homogeneous medium has been considered for simulations so that the target/ROI volume ratio is a good estimate of the site-hit probability. However, the relative macroscopic cross sections (or interaction probability per unit path length) should be accounted for in a model with regions of different chemical composition. That is, a region with a relatively high cross section would appear larger and would receive a relatively higher number of events. Our results seem to conflict with the ones reported by Semsarha et al. [17], who found higher site-hit probabilities for smaller target sizes. However, they defined the hit probability as the ratio of the number of hits within the strands to the ones occurring within the whole DNA volume. According to our understanding, this probability should be equal to the ratio of the volume occupied by the sugar-phosphate groups plus the hydration shell to the volume of the whole DNA cylinder. We have calculated this ratio for the dimensions shown in Figure 2 of [17] and found values of 0.85, 0.81, and 0.71 for the A-, B-, and Z-DNA conformations. These results are similar to the values shown in Table 5 of [17], except for the B-DNA case. These discrepancies should be further investigated.

**Figure 5:**
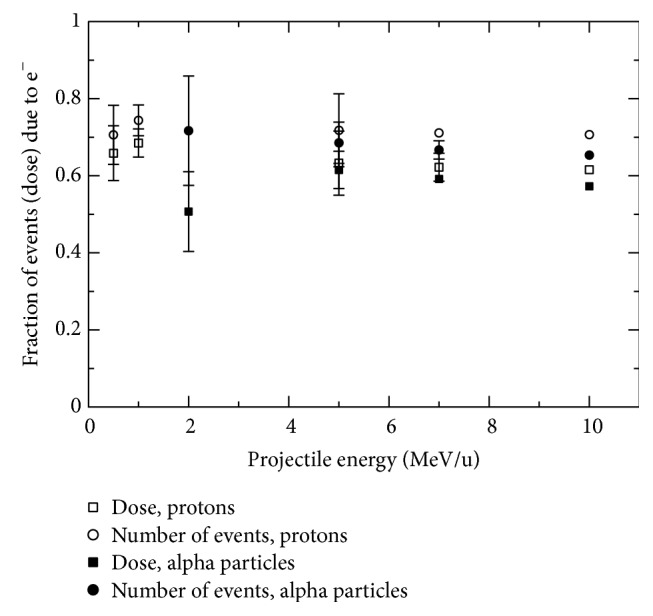
Fraction of the number of inelastic events and absorbed dose due to secondary electrons produced by protons and alpha particles.

Finally, Figure 5 shows the fraction of the number of inelastic events and absorbed dose involving the secondary electrons produced by protons and alpha particles. Approximately 70% of all the inelastic collisions and ~60% of the absorbed dose are due to secondary electrons. The fraction of events and the absorbed dose due to secondary electrons for protons are slightly higher than for alpha particles at the same incident energy. This result could be explained by the fact that protons produce electrons with higher average energy than alpha particles at the same ion energy. Protons are lighter than alpha particles and thus can transfer more energy, on average, to bound electrons than alpha particles with the same energy. The values shown in this figure depend weakly on the particle type and energy (or LET). The fraction of dose and number of inelastic events due to secondary electrons are within 0.61 ± 0.05 and 0.70 ± 0.03, respectively, for particle LET values ranging from ~4.8 to ~235.0 keV/*μ*m.

## 4. Conclusions

The direct DSB yield at the same LET is higher for the lightest ion of the ones studied in this work. Thus, it can be inferred that the lighter the ion the higher its capacity to produced spatially dense ionizations for a given LET. This result has been recently confirmed by clustering analysis for ions as heavy as carbon [24].

The site-hit probability is determined by the effective (union) volume occupied by the DNA targets to the first order. However, there is an additional increase in this probability due to the clustered nature of the energy deposition events. This volume could depend on the DNA configuration. In this work and for practical reasons, different numbers of base pairs were used for each DNA configuration, but this number should remain constant during the cell cycle, independently of the DNA configuration. Thus, the total volume occupied by targets would be determined by a single target volume, which would depend on the DNA configuration. As it is expected that the A-, B-, and Z-DNA conformations have similar sugar-phosphate group volumes, their site-hit probabilities should be similar for them if the number of base pairs included in the geometrical model is the same.

The strand break yield can be determined by multiplying the site-hit probability times the number of inelastic events per unit absorbed dose and dividing this result by the number of base pairs contained in the model. Then, as the site-hit probability is highly influenced by the effective volume of the sugar-phosphate groups, the direct total strand break yield for a given DNA form is determined almost entirely by the number of inelastic events per unit absorbed dose. By the way, this number depends weakly on the beam properties, as shown previously [19]. In addition, the fraction of the number of inelastic events and of the total energy deposited when ions impact on water is quasi-independent of the incident particle energy but depends on the particle type. The correlation shown by the last two quantities is explained by the weak variation of the mean energy deposit per event when charged particles interact with matter [26].

It was also found that the DSB yield increases with the linear density of base pairs along the DNA helix, which is determined in this work by the number of bp per nucleosome. Thus, the DSB yield is importantly influenced by the DNA conformation.

It is expected that this work will help better understand the physical aspects of the early stages of DNA damage induction by ionizing particles.

## Figures and Tables

**Table 1:** Dimensions of the main DNA structures for the three configurations studied in this work. Most of these data were extracted from [7]. The target volumes were determined according to these dimensions and the geometrical model used (see [18] for details). All dimensions are shown in nm, unless otherwise stated.

Features	DNA configurations		
	A	B	Z
Helix orientation	Right-handed	Right-handed	Left-handed
DNA diameter	2.55	2.37	1.84
bp diameter	1.0	1.0	0.6
bp axial step	0.23	0.33	0.38
Helix pitch	2.46	3.2	3.0
bp/helix turn	10.7	10	12
bp/nucleosome	286	198	172
Helix axial shift	0.76	1.2	0.77
Target angular aperture	87°	73°	60°
Target height	0.119	0.183	0.249
Total number of bp	7.72 × 10^8^	5.35 × 10^8^	4.64 × 10^8^
Target volume (nm^3^)	0.12	0.13	0.10

**Table 2:** Average site-hit probability for each DNA configuration-particle combination. Corresponding values predicted from the volume ratio defined in the text are also shown.

DNA configuration	Protons	Alpha part.	Pred. value
A	0.040 ± 0.002	0.040 ± 0.004	0.038
B	0.030 ± 0.001	0.030 ± 0.003	0.029
Z	0.0188 ± 0.0008	0.0194 ± 0.002	0.018
